# Dopamine and prolactin in migraine: Mechanisms and potential therapeutic targets

**DOI:** 10.1111/jne.70098

**Published:** 2025-10-06

**Authors:** Astrid Johannesson Hjelholt, Randi Maria Hanghøj Tei, Hans Christoph Diener, Jens Otto Lunde Jørgensen

**Affiliations:** ^1^ Department of Endocrinology and Internal Medicine Aarhus University Hospital Aarhus Denmark; ^2^ Department of Clinical Medicine Aarhus University Aarhus Denmark; ^3^ Department of Clinical Pharmacology Aarhus University Hospital Aarhus Denmark; ^4^ Department of Neuroepidemiology, Institute for Medical Informatics, Biometry and Epidemiology, Faculty of Medicine University Duisburg‐Essen Essen Germany

**Keywords:** dopamine, dopamine receptor agonists, migraine, prolactin

## Abstract

Migraine is a complex neurovascular disorder characterized by activation and sensitization of the trigeminovascular system. Hyperprolactinemia is associated with headache, and improvement following prolactin‐lowering therapy has been reported in observational studies. Preclinical evidence indicates that prolactin promotes neuronal excitability and sensitization within trigeminal pathways, particularly in females. Downregulation of the protective long prolactin receptor isoform further increases susceptibility to migraine‐relevant triggers. Prolactin secretion is under tonic inhibition by dopamine, a key hypothalamic regulator that also modulates central pain pathways. The role of dopamine in migraine pathophysiology is complex. On one hand, prodromal symptoms such as nausea and yawning are considered dopamine‐mediated. On the other hand, experimental studies show that dopamine directly inhibits nociceptive trigeminovascular activity in addition to lowering prolactin. Dopamine receptor agonists are established treatments for hyperprolactinemia and have demonstrated a positive effect on hyperprolactinemia‐associated headache. A recent placebo‐controlled randomized clinical trial suggests that dopamine agonist treatment can be used as a preventive migraine treatment. In conclusion, prolactin and dopamine may modulate migraine via distinct but converging neuroendocrine pathways, which could represent targets for migraine prevention.

## Graphical abstract

Prolactin and dopamine modulate migraine‐relevant trigeminal pathways. Hyperprolactinemia is associated with migraine‐like headache, and preclinical studies show that prolactin enhances trigeminal excitability, particularly in females. Dopamine inhibits prolactin release and may also directly suppress trigeminovascular activity via D2 receptors in the trigeminocervical complex. Dopamine receptor agonists such as cabergoline, used to treat hyperprolactinemia, have shown beneficial effects in prolactin‐related headache and may reduce migraine burden.

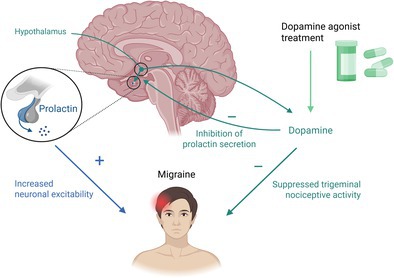

## 1 INTRODUCTION

Migraine is a neurovascular disorder characterized by recurrent attacks of moderate to severe, unilateral, and pulsating headaches lasting 4–72 h. Migraine headache is frequently accompanied by nausea, photo‐, and phonophobia and may be preceded by transient neurological symptoms known as aura.^1^ Many patients also experience prodromal and postdromal symptoms, including changes in appetite and sleep regulation, yawning, sensory hypersensitivity, and mood changes.^2^

The estimated global lifetime prevalence of migraine approximates 17.5%^3^ and migraine ranks as the second leading cause of years lived with disability.^4^ While migraine prevalence is comparable in boys and girls before puberty, it increases significantly in females after menarche, affecting ~30% of women between 30 and 50 years of age, which is three to four times the prevalence observed in men.^5^ This striking difference suggests a role for sex hormone modulation of migraine pathways, and 20%–25% of women with migraine have attacks mainly in the perimenstrual period.^6^

**FIGURE 1:**
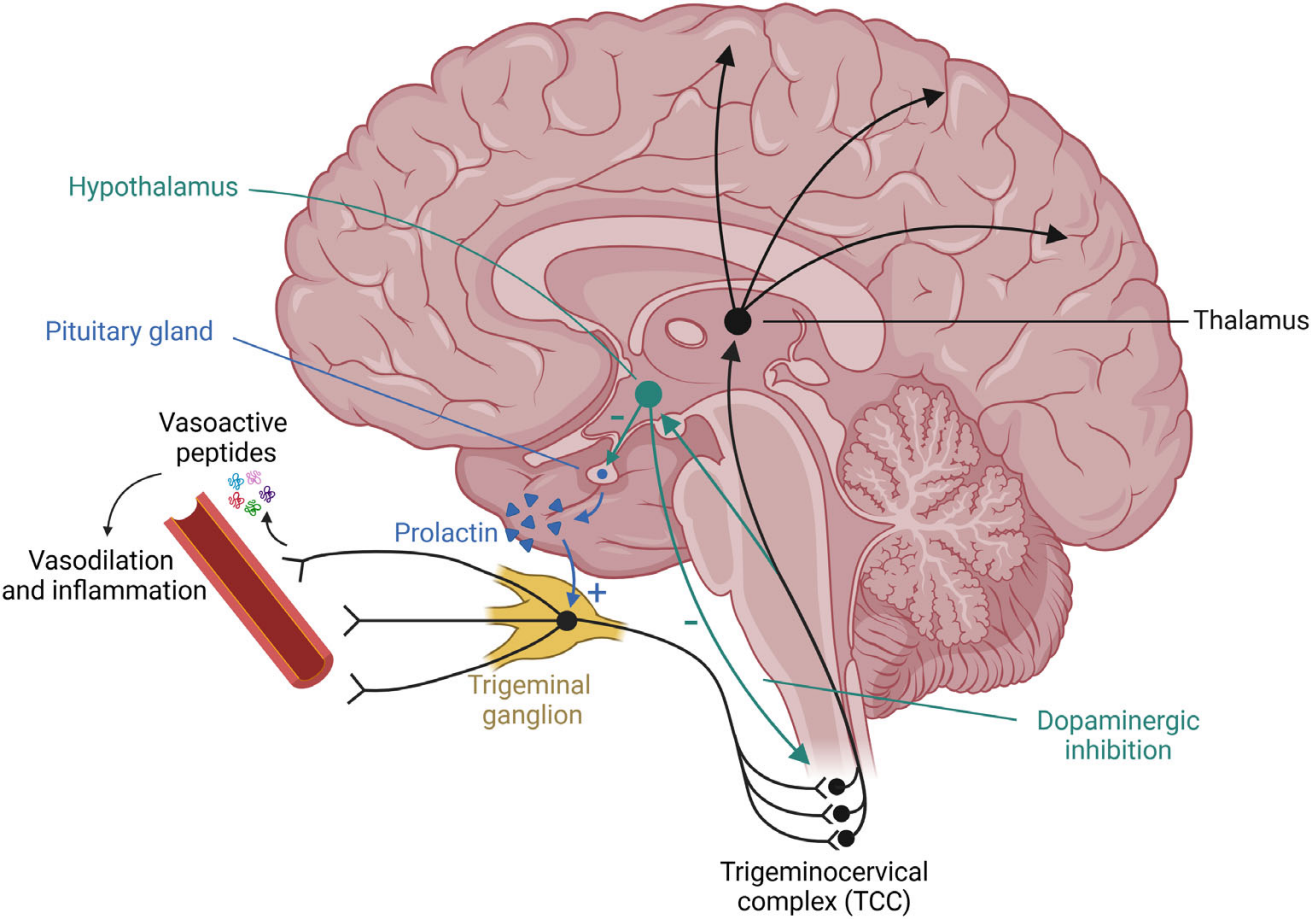
Neuroendocrine modulation of trigeminovascular migraine pathways by prolactin and dopamine. Trigeminal afferents originating in the trigeminal ganglion release vasoactive peptides that induce meningeal vasodilation and inflammation. Ascending signals are relayed via the trigeminocervical complex (TCC) to the thalamus and higher cortical areas. Dopaminergic projections (green) from the hypothalamic A11 nucleus provide inhibitory input to second‐order neurons within the TCC, modulating central sensitization. Prolactin (blue), secreted by the anterior pituitary under tonic inhibition by hypothalamic dopamine, sensitizes trigeminal neurons and enhances nociceptive transmission.

Central to migraine pathogenesis is the trigeminovascular system, which comprises trigeminal nerve afferents innervating the meninges and associated blood vessels. In preclinical models, activation of first‐order nociceptive neurons in the trigeminal ganglion releases vasoactive neuropeptides, including calcitonin gene‐related peptide (CGRP) and pituitary adenylate cyclase‐activating polypeptide (PACAP), promoting vasodilation and neurogenic inflammation (Figure 1).^1^, ^7^ These signals are conveyed to second‐order neurons in the trigeminocervical complex (TCC) within the trigeminal nucleus caudalis. With repeated or sustained stimulation, neurons in the TCC become sensitized, leading to exaggerated responses to both noxious and innocuous stimuli. From the TCC, projections ascend to third‐order neurons in the thalamus, which transmit nociceptive information to cortical regions involved in pain perception, including the somatosensory cortex.^1^

Preventive migraine therapy, recommended for patients with frequent or disabling attacks, has traditionally included antihypertensives, tricyclic antidepressants, anticonvulsants, and calcium channel blockers.^1^ More recently, therapies targeting the CGRP pathway have been introduced, which include monoclonal antibodies and small molecule receptor antagonists, the so‐called gepants.^8^ Acute treatment comprises nonsteroidal anti‐inflammatory drugs (NSAIDs), triptans, and gepants, which are also approved for acute use.^1^, ^8^ The serotonergic 5‐HT_1_B/_1_D receptor agonists, including triptans and ergot derivatives, act not only through vasoconstriction but also by inhibiting CGRP release from trigeminal sensory fibers.^9^, ^10^

The hypothalamus controls a wide range of physiological functions, including hormonal regulation, circadian rhythms, autonomic output, and integration of stress responses. Anatomically and functionally, the hypothalamus is connected to several migraine‐relevant brain regions, including the TCC.^11^, ^12^ Individuals with migraine often experience disturbances in sleep and autonomic function,^13^ and functional imaging studies demonstrate hypothalamic activation during both the premonitory and the headache phase of migraine attacks, supporting a role in the initiation and propagation of migraine symptoms.^14^, ^15^ Prolactin, which is under tonic inhibition by hypothalamic dopamine, has been implicated in migraine pathophysiology through its modulatory effects on trigeminal pain processing.^16^, ^17^

This review examines the role of dopamine and prolactin in migraine pathophysiology, with special emphasis on potential clinical therapeutic implications.

## 2 THE ROLE OF PROLACTIN IN MIGRAINE PATHOPHYSIOLOGY

Prolactin is a peptide hormone secreted by lactotrophs in the anterior pituitary.^18^ Under physiological conditions, prolactin is released in a pulsatile manner following a circadian rhythm with nocturnal surges after sleep onset. Basal secretion is predominantly regulated by tonic inhibition by dopamine via D₂ receptors in the tuberoinfundibular pathway. Several factors stimulate prolactin release including estrogen, thyrotropin‐releasing hormone (TRH), serotonin, and vasoactive intestinal peptide.^11^ An estrogen‐mediated stimulation of lactotroph proliferation and prolactin secretion is observed during pregnancy and lactation, where hypothalamic dopamine production is downregulated.^18^

**FIGURE 2:**
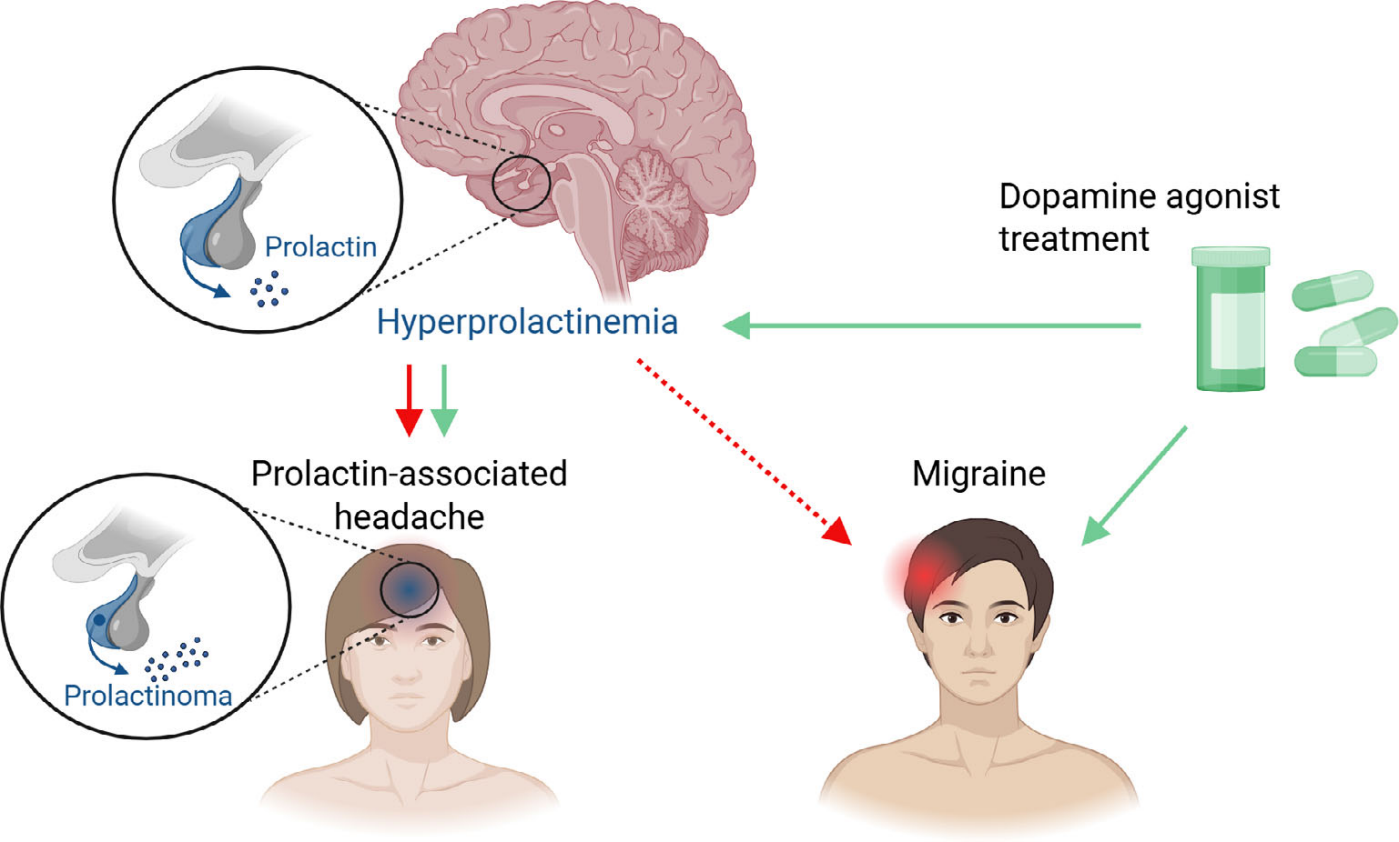
Hyperprolactinemia‐associated headache and migraine. Hyperprolactinemia, most commonly caused by a prolactin‐secreting pituitary adenoma (prolactinoma), is frequently associated with headache (red arrow). The clinical phenotype often resembles migraine and may occur even in the absence of pituitary enlargement or structural invasion. Elevated prolactin levels have also been reported in individuals with migraine without prolactinoma (red dotted arrow). Dopamine receptor agonist treatment, which lowers prolactin levels, has been associated with headache improvement independent of tumor size reduction and may also reduce migraine burden in patients without overt hyperprolactinemia (green arrows).

The biological actions of prolactin are mediated by the prolactin receptor (PRLR) of which three human isoforms have been identified—long (PRLR‐L), intermediate (PRLR‐I), and short (PRLR‐S)—which differ in the length of the intracellular domain.^19^ While best known for its role in lactation, prolactin exerts a broad range of neuroendocrine and immunomodulatory effects in both sexes, influencing immune responses, behavior, energy metabolism, and central neuronal activity.^18^ PRLR expression is also present in nociceptive structures, and functional studies indicate that prolactin modulates pain signaling pathways.^18^ Clinical studies report an association between migraine and hyperprolactinemia (Figure 2),^19^ which fits with preclinical evidence of a mechanistic link between prolactin signaling and trigeminal nociception.^20^ In addition, prolactin has been reported to activate immune cells,^18^ which may in turn contribute to inflammatory mechanisms in the meninges relevant to migraine.

### 2.1 Hyperprolactinemia and migraine

Elevated serum prolactin levels have been reported in individuals with migraine across multiple clinical studies.^19^ A recent systematic review and meta‐analysis of 15 case–control comparisons, including 460 patients with migraine and 429 healthy controls, confirmed significantly higher circulating prolactin concentrations in patients with migraine,^21^ but unchanged or even reduced prolactin levels have also been reported.^22^, ^23^, ^24^ Increased prolactin has been observed during acute headache attacks^25^ and observational studies indicate that prolactin may be elevated in patients with chronic migraine.^26^, ^27^, ^28^

Hyperprolactinemia is more commonly observed in females and is usually caused by a pituitary prolactinoma.^29^ Headache is frequent in patients with hyperprolactinemia^19^, ^30^, ^31^, ^32^, ^33^, ^34^, ^35^, ^36^, ^37^, ^38^ and although factors such as increased intrasellar pressure and invasion of pain‐sensitive structures caused by the pituitary adenoma may contribute to headache, several lines of evidence suggest a direct role of prolactin in headache pathophysiology. Headache is more common in patients with prolactinomas compared with those with nonfunctioning pituitary adenomas,^30^ and the clinical phenotype often resembles migraine headache.^31^, ^34^, ^38^ Tumor size or invasiveness does not consistently correlate with headache presence and severity^32^, ^33^ and individuals with hyperprolactinemia in the absence of pituitary enlargement also report increased headache frequency.^25^, ^32^, ^35^, ^36^ Importantly, treatment of hyperprolactinemia with dopamine receptor agonists has been associated with significant headache relief, even in the absence of pituitary tumor shrinkage (Figure 2).^31^, ^32^, ^35^

### 2.2 Prolactin‐mediated trigeminal modulation: Mechanistic and sex‐specific insights

Experimental models reveal that prolactin facilitates trigeminal nociception and migraine‐like behaviors in a sex‐dependent manner, primarily through activation of PRLR expressed on trigeminal sensory neurons, with current evidence indicating that this sex difference is limited to peripheral rather than central circuits (Figure 1).^16^, ^17^, ^39^ In female rodents, prolactin enhances CGRP‐induced responses and dural activation, effects that are attenuated by disrupting either pathway, underscoring bidirectional crosstalk between prolactin and CGRP signaling.^40^

In situ hybridization and immunohistochemical studies in rodent and human tissue demonstrate that PRLR‐S is predominantly expressed on trigeminal sensory neurons, whereas PRLR‐L is mainly localized to glial cells with limited neuronal expression.^41^ Activation of PRLR‐S has been shown to increase neuronal excitability and promote migraine‐like behaviors, whereas PRLR‐L exerts an inhibitory effect.^39^ Indeed, PRLR‐L overexpression in sensory neurons attenuates pain sensation, while deletion promotes mechanical allodynia in female mice.^39^

In a mouse model of medication overuse headache, repeated sumatriptan administration induces cutaneous allodynia in both sexes, but only the female mice exhibit downregulation of PRLR‐L expression.^41^, ^42^ This favors PRLR‐S–mediated signaling and increases vulnerability to migraine‐like pain. Notably, treatment with the dopamine receptor agonist cabergoline prevented PRLR‐L downregulation in female animals.^42^, ^43^ Collectively, these findings support a prolactin‐dependent mechanism that increases migraine susceptibility. This involves downregulation of the inhibitory PRLR‐L isoform and enhanced PRLR‐S signaling in trigeminal sensory neurons and is suppressed by dopamine agonist treatment.

## 3 DOPAMINERGIC MECHANISMS IN MIGRAINE PATHOPHYSIOLOGY

Dopamine is a catecholaminergic neurotransmitter involved in locomotor control, reward, cognition, and neuroendocrine signaling. Dopamine receptors are grouped into D1‐like and D2‐like families, which exert opposing effects on intracellular cyclic AMP levels.^44^ In the mammalian brain, dopamine‐producing neurons in the midbrain, hypothalamus, and olfactory bulb give rise to the main dopaminergic pathways: the nigrostriatal, mesolimbic, mesocortical, and tuberoinfundibular systems.^45^

Emerging evidence supports a complex, context‐dependent role for dopamine in migraine pathophysiology. Yawning, somnolence, and nausea are premonitory migraine symptoms considered to reflect dopaminergic activation.^44^, ^46^ It is also suggested that migraine patients may exhibit dopaminergic hypersensitivity,^47^, ^48^, ^49^, ^50^ and dopamine receptor antagonists are used to treat migraine‐associated nausea and have been reported to attenuate headache intensity.^51^, ^52^, ^53^, ^54^, ^55^, ^56^, ^57^ The underlying evidence level, however, is low.^44^

Conversely, preclinical studies indicate that dopamine exerts an inhibitory effect on nociceptive pathways in migraine (Figure 1).^11^, ^44^, ^46^ Immunohistochemical studies demonstrate D2 receptors on neurons within the TCC, indicating direct postsynaptic modulation,^58^ and data from rodent models show that stimulation of the hypothalamic A11 nucleus inhibits dural‐evoked responses in second‐order neurons of the TCC. This effect is reversed by D2 receptor antagonists, and lesioning of the A11 nucleus enhances neuronal responses to both noxious and innocuous stimuli, indicating a tonic dopaminergic inhibitory influence (Figure 1).^59^ Consistent with this, D2 receptor activation inhibits GluA1‐containing AMPA receptors and suppresses central sensitization within the TCC, supporting an inhibitory role of dopamine in migraine pathophysiology.^60^ Additional evidence suggests that dopaminergic and serotonergic systems converge within the TCC where co‐expression of D2 and 5‐HT_1_B/_1_D receptors has been demonstrated. Activation of these receptors suppresses trigeminal nociceptive activity, which is abrogated by antagonism of either receptor type.^61^

## 4 DOPAMINE RECEPTOR AGONISTS IN THE TREATMENT OF MIGRAINE

Dopamine agonists such as bromocriptine and cabergoline are commonly used in the treatment of hyperprolactinemia.^62^ Both are ergot derivatives with high affinity for D_2_‐like receptors. Cabergoline also binds to serotonergic 5‐HT_1_B/_1_D receptors and is distinguished by favorable pharmacokinetics that allow for once‐weekly oral dosing. It is generally better tolerated than other agents in its class.^62^, ^63^

Clinical reports indicate that dopamine receptor agonists have beneficial effects in patients with prolactin‐associated headache, including migraine.^25^, ^26^, ^31^, ^32^, ^35^ In patients with prolactinoma, treatment led to complete or partial headache resolution in a substantial proportion of cases.^31^, ^32^, ^35^ In chronic migraine with concomitant hyperprolactinemia, cabergoline reduced attack frequency,^26^ and prolactin‐provoked migraine attacks respond to dopamine agonists in patients with macroprolactinoma.^25^ In an open‐label prospective study targeting menstrual migraine without known prolactin abnormalities, bromocriptine reduced attack frequency by 72% after 1 year in 18 of 24 women.^64^ Moreover, in an older placebo‐controlled trial, the ergot‐derived dopamine and serotonin receptor agonist lisuride hydrogen maleate significantly reduced migraine attack frequency.^65^ Collectively, these findings support the efficacy of dopamine receptor agonists in patients with hyperprolactinemia‐associated headache and suggest a potential benefit also in migraine patients without hyperprolactinemia.

**FIGURE 3:**
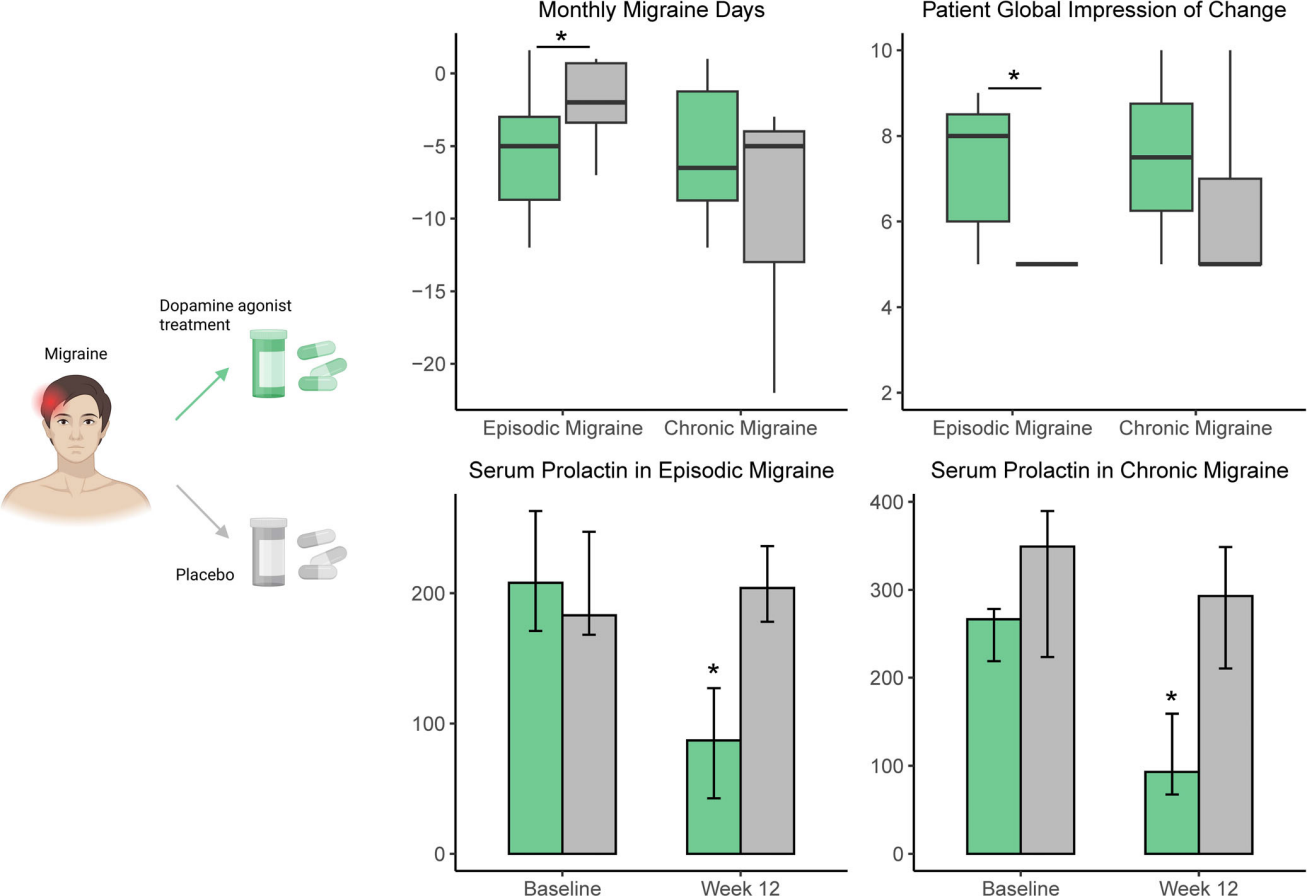
Preventive effect of cabergoline in migraine. In a randomized, placebo‐controlled pilot study, cabergoline 0.5 mg once weekly for 12 weeks reduced monthly migraine days and improved Patient's Global Impression of Change (PGIC) compared with placebo in participants with episodic migraine. No significant effect was observed in chronic migraine. Serum prolactin levels declined after cabergoline in both participants with episodic and chronic migraine. Reduction in monthly migraine days was defined as the change from baseline to the final 28 days of the treatment period. Participants rated their overall change in disease status using the single‐item PGIC scale (1 = very much worse, 5 = no change, 10 = very much improved). Medians, percentiles, minimums, and maximums are presented. *p < .05.

Motivated by these findings, we recently conducted a randomized, placebo‐controlled pilot study to evaluate cabergoline as a preventive treatment for migraine.^28^ In 20 participants with episodic migraine, cabergoline 0.5 mg once weekly for 12 weeks as add‐on therapy reduced monthly migraine days by 3.6 days compared with placebo. This reduction seems clinically meaningful, as recent estimates show a mean decrease of 1.9 monthly migraine days with anti‐CGRP treatments in episodic migraine, compared with 0.9–1.7 days with other preventive drugs such as propranolol, topiramate, valproate, and amitriptyline.^66^ Although the reduction in migraine days was not significant among the 13 participants with chronic migraine, the overall patient‐reported improvement (Patients' Global Impression of Change, PGIC) was significantly greater following cabergoline treatment in the full study population^28^ (Figure 3).

Given preclinical and clinical evidence that prolactin signaling and dopamine agonist treatment may be particularly relevant in females, sex differences should be considered when interpreting treatment effects. In our pilot study, only three male participants were included. One received cabergoline, with no observed reduction in migraine days. The predominance of female participants limits generalizability, and caution is warranted when applying the findings to males.^28^

All the participants had serum prolactin levels within the normal range at baseline, though higher levels were found in chronic migraine. Cabergoline significantly decreased prolactin levels in all participants, but this was not correlated to the reduction in migraine days.^28^ Although this indicates that cabergoline may be efficient in the prevention of migraine independently of hyperprolactinemia, the small sample size is a limitation, and larger randomized, controlled trials are needed to confirm the findings.

## 5 CONCLUDING REMARKS

Clinical and preclinical findings highlight a neuroendocrine link between dopamine, prolactin, and migraine pathophysiology. Prolactin enhances neuronal excitability and increases susceptibility to migraine triggers—particularly in females—via actions on trigeminal sensory neurons. Clinical reports show an association between hyperprolactinemia and headache. Dopamine agonists may therefore reduce migraine frequency and severity through two distinct mechanisms, one of which involves prolactin lowering and reduced trigeminal sensitization, and the other mediated by dopaminergic modulation of trigeminovascular signaling. Consistent with this, dopamine receptor agonists have demonstrated beneficial effects in case reports and observational studies of patients with hyperprolactinemia‐associated headache and menstrual migraine, and more recently in a randomized, placebo‐controlled trial in individuals with episodic migraine.

Taken together, these findings support a pathophysiological and therapeutic role for prolactin and dopamine signaling in migraine. Repurposing dopamine receptor agonists to target these pathways may offer a cost‐effective and accessible strategy for migraine prevention, particularly in a field where high drug costs remain a major barrier.^67^ Further clinical studies and basic research are needed to replicate the beneficial effects and elucidate the underlying mechanisms.

## AUTHOR CONTRIBUTIONS

**Astrid Johannesson Hjelholt:** Visualization; conceptualization; writing – original draft; writing – review and editing. **Randi Maria Hanghøj Tei:** Visualization; writing – review and editing. **Hans Christoph Diener:** Supervision; writing – review and editing. **Jens Otto Lunde Jørgensen:** Conceptualization; writing – review and editing; supervision.

## FUNDING INFORMATION

The authors received no specific funding for this work.

## CONFLICT OF INTEREST STATEMENT

The authors declare no conflicts of interest.

## Data Availability

Data sharing not applicable to this article as no datasets were generated or analysed during the current study.
